# Effective biodegradation of chicken feather waste by co-cultivation of keratinase producing strains

**DOI:** 10.1186/s12934-019-1134-9

**Published:** 2019-05-18

**Authors:** Zheng Peng, Xinzhe Mao, Juan Zhang, Guocheng Du, Jian Chen

**Affiliations:** 1 School of Biotechnology, Jiangnan University, 1800 Lihu Road, Wuxi, 214122 China; 2 National Engineering Laboratory for Cereal Fermentation Technology, Jiangnan University, 1800 Lihu Road, Wuxi, 214122 China; 3 Key Laboratory of Industrial Biotechnology, Ministry of Education, Jiangnan University, 1800 Lihu Road, Wuxi, 214122 China

**Keywords:** Chicken feather waste, Biodegradation, Cocultivation, Laboratory scale fermenter, Hydrolysate

## Abstract

**Background:**

Chicken feather, a byproduct of poultry-processing industries, are considered a potential high-quality protein supplement owing to their crude protein content of more than 85%. Nonetheless, chicken feathers have been classified as waste because of the lack of effective recycling methods. In our previous studies, *Bacillus licheniformis* BBE11-1 and *Stenotrophomonas maltophilia* BBE11-1 have been shown to have feather-degrading capabilities in the qualitative phase. To efficiently recycle chicken feather waste, in this study, we investigated the characteristics of feather degradation by *B. licheniformis* BBE11-1 and *S. maltophilia* BBE11-1. In addition, in an analysis of the respective advantages of the two degradation systems, cocultivation was found to improve the efficiency of chicken feather waste degradation.

**Results:**

*B. licheniformis* BBE11-1 and *S. maltophilia* BBE11-1 were used to degrade 50 g/L chicken feather waste in batches, and the degradation rates were 35.4% and 22.8% in 96 h, respectively. The degradation rate of the coculture system reached 55.2% because of higher keratinase and protease activities. Furthermore, cocultivation was conducted in a 3 L fermenter by integrating dissolved oxygen control and a two-stage temperature control strategy. Thus, the degradation rate was greatly increased to 81.8%, and the conversion rate was 70.0% in 48 h. The hydrolysates exhibited antioxidant activity and contained large quantities of amino acids (895.89 mg/L) and soluble peptides.

**Conclusions:**

Cocultivation of *B. licheniformis* BBE11-1 and *S. maltophilia* BBE11-1 can efficiently degrade 50 g/L chicken feather waste and produce large amounts of amino acids and antioxidant substances at a conversion rate of 70.0%.

**Electronic supplementary material:**

The online version of this article (10.1186/s12934-019-1134-9) contains supplementary material, which is available to authorized users.

## Background

Owing to the high consumption of poultry products, millions of tons of chicken feathers are produced each year worldwide [1, 2]. The majority of these feathers are discarded or burned as waste, while a small proportion is used in down products and insulation materials [3]. Chicken feathers contain more than 85% of crude protein, 70% of amino acids, high-value elements, vitamins, and growth factors [4]. Researchers have shown great interest in applying these materials to various products such as feed [5], fertilizer [6], and biofilm [7], etc., chicken feathers have high mechanical stability and are not easily hydrolyzed by common proteolytic enzymes.

Chicken feathers have stable structures because of the large abundance of the rigid protein keratin. Keratin is a fibrous structural protein present in the epidermis and epidermal appendages of vertebrates, such as feathers, skin and nails, and is rich in cysteine residues and disulfide bonds [8, 9]. Disulfide bonds can create cross-links among protein peptide chains, thereby generating a dense polymeric structure in conjunction with hydrogen bonding and hydrophobic forces. Therefore, keratin is quite stable with high mechanical strength [10]. Chicken feathers are degraded mainly by physical methods (pressurized hydrolysis, and puffing) and chemical methods (acid and alkali) [11–13]. However, these methods have limitations such as high energy consumption during the production process and substantial of damage to the products [14]. In recent years, biotechnological methods have been used to degrade keratin. Microbial processes are not only environmentally friendly [15], but also maintain the original structure and activity of the products [16].

Currently, studies on biodegradation are focused on the screening and identification of microorganisms that can degrade feathers (e.g., bacteria and fungi) [17–19]. Additionally, purification strategies, enzymatic properties, and heterologous expression of keratinase have also been reported [20, 21]. Nevertheless, few studies have examined the biodegradation of keratin in intact chicken feathers because of the complex structure of keratinous waste and the difficultly in degrading chicken feathers (≥ 50 g/L). One study reported that *Bacillus* sp. C4 degrades only 75% of a 5% (w/v) suspension of chicken feathers in 8 days in a time-consuming and low-efficiency process [22]. Additionally, scaling up the biodegradation process in a fermenter is challenging, but essential for industrial applications. To date, only recombinant *Bacillus subtilis* DB 100 (p5.2) culture has been scaled up to a 14 L Bio Flo 110 fermenter to achieve nearly complete degradation of 2% (w/v) chicken feathers [23]. Therefore, it is necessary to develop an effective biodegradation process in a fermenter with chicken feathers as a substrate.

The products of keratinase hydrolysis of feathers are mainly amino acids and soluble peptides and display antioxidant properties [24, 25]. Antioxidants are important molecules that provide protection against free radicals or scavenge free radicals and are essential in humans and animals [26]. Antioxidants derived from plants and animals or antioxidant polypeptides obtained by decomposing natural proteins are more widely used than chemically synthesized antioxidants. Discarded chicken feathers are a large potential source of proteins and antioxidant peptides [27].

We have previously identified two keratinolytic strains, *Bacillus licheniformis* BBE11-1 [28] and *Stenotrophomonas maltophilia* BBE11-1 [29]. Both strains can hydrolyze feathers, but their growth and enzyme production conditions are quite different, and they are not suitable for the degradation of a large quantities of feathers. In addition, the use of keratinase alone does not hydrolyze feathers. In this study, we cultured these two strains individually or together in a 3 L fermenter with a large amount of the substrate. Employing an integrated and innovative biotechnological process efficient degradation of chicken feathers was achieved and this technique can be further used to isolate bioactive compounds such as antioxidant peptides and amino acids proving to be important for industrial biodegradation.

## Results and discussion

### Degradation effects of stand-alone and cocultured strains in shake flasks

**Fig. 1:**
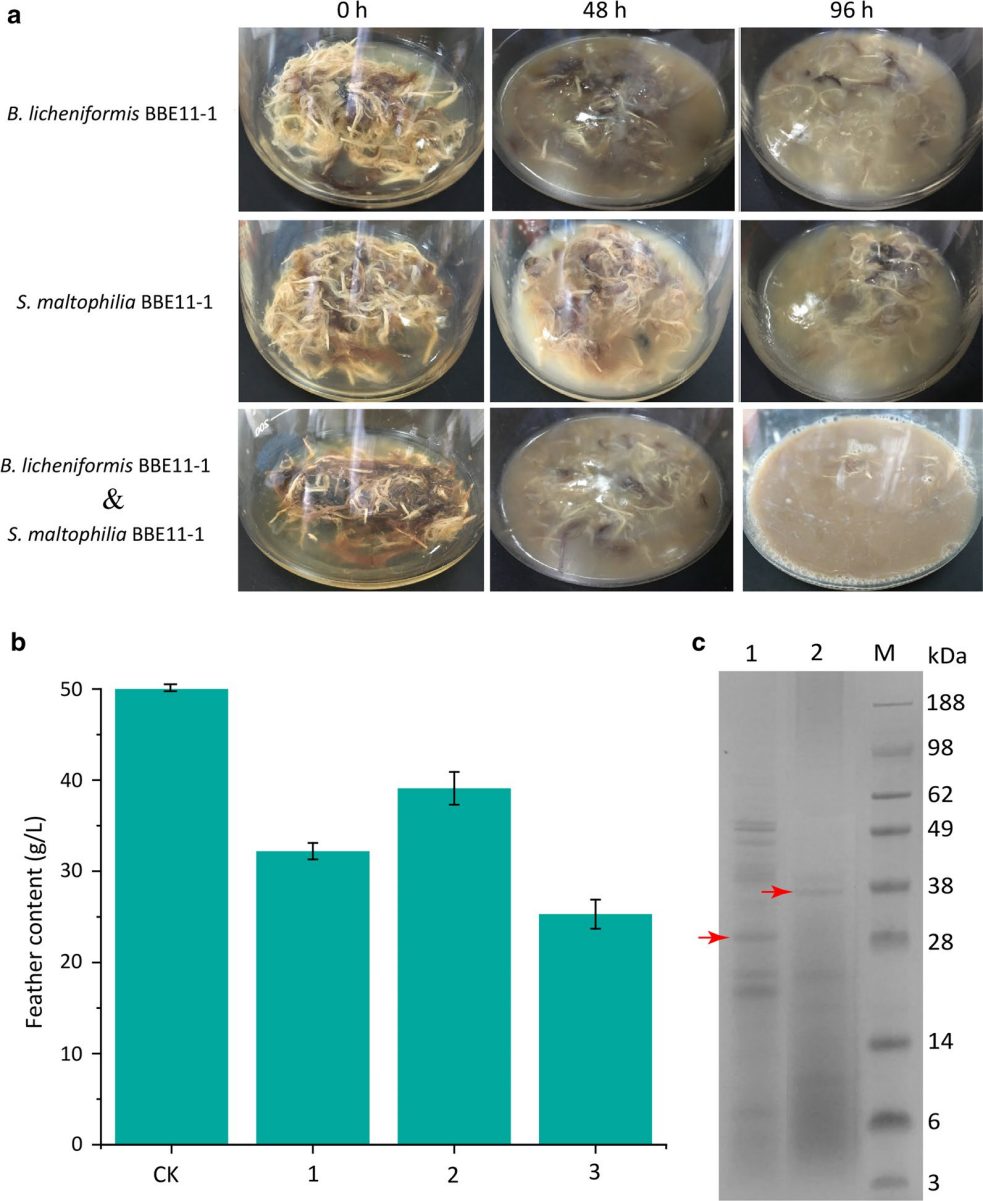
Shaking flask experiments. **a** Chicken feather degradation efficiency when inoculated with *B. licheniformis* BBE11-1 or *S. maltophilia* BBE11-1. **b** The change in dry weight of chicken feathers. 1: Inoculated with *B. licheniformis* BBE11-1; 2: inoculated with *S. maltophilia* BBE11-1; 3: inoculated with *B. licheniformis* BBE11-1 and *S. maltophilia* BBE11-1. **c** A zymogram of the degradation system. 1: The degradation system of *B. licheniformis* BBE11-1; 2: the degradation system of *S. maltophilia* BBE11-1

Previous studies have shown that both *B. licheniformis* BBE11-1 and *S. maltophilia* BBE11-1 can decompose 10 g/L chicken feathers but have different keratinase activities [28]. In this study, a system was designed to degrade 50 g/L feathers, which is the upper limit for shake flasks and fermenters. As presented in Fig. 1a, b, only 22.8% of chicken feathers were hydrolyzed after incubation with *S. maltophilia* BBE11-1 at 23 °C and 220 rpm for 96 h, but the degradation rate increased to 35.4% after incubation with *B. licheniformis* BBE11-1 at 37 °C and 220 rpm for 96 h. This finding is consistent with the results of other studies indicating incomplete degradation of large amounts of chicken feathers in a short period of time [30–32]. SDS-PAGE of the fermentation broth was conducted to analyze the differences in chicken feather degradation ability between the two strains. Figure 1c (the bands indicated by the arrows are keratinase) indicates that the enzymolytic system of *B. licheniformis* BBE11-1 has more kinds of enzymes than that of *S. maltophilia* BBE11-1. This situation may explain why *B. licheniformis* BBE11-1 had a better ability to hydrolyze chicken feathers: because the hydrolysis of feathers is a keratinase-based multienzyme synergistic process [33, 34].

The degradation system of *S. maltophilia* BBE11-1 showed higher keratinase activity, while the degradation system of *B. licheniformis* BBE11-1 was more abundant in enzymes. Therefore, it was hypothesized that combining the two systems could improve the efficiency of chicken feather degradation. The coculture system was based on a temperature conversion strategy, 37 °C to 30 °C, in the first 12 h of incubation conducted at 37 °C for rapid cell growth. As depicted in Fig. 1a, b, the degradation rate of chicken feathers in the coculture system was significantly improved. After 10% inoculation for 96 h incubation, the dry weight diminished by approximately 50% (25.4 g/L). This result indicates that a coculture of two bacterial strains (each possessing chicken feather-degradation ability) was more efficient for degrading large amounts of chicken feathers.

### Optimization of coculture conditions

As illustrated in Additional file 1: Fig. S1a, the coculture system degraded more than a half of the feathers (55.2%) and manifested the highest keratinase activity (244.5 U/mL) at initial pH of 7. The corresponding degradation efficiency and keratinase activity diminished as initial pH was increased [16]. *B. licheniformis* BBE11-1 and *S. maltophilia* BBE11-1 were inoculated (volume of each strain was 10% of the total sample volume) in the optimum ratio (1:1) to achieve the best degradation (48.1%) and the highest keratinase activity (138.2 U/mL; Additional file 1: Fig. S1b). Increasing the inoculum volume of *B. licheniformis* BBE11-1 or *S. maltophilia* BBE11-1 did not further promote degradation. This phenomenon might be related to the growth relationship between the two bacteria in the coculture system and their ability to produce enzymes.

Finally, optimization of the second-stage transition temperature was carried out based on the determination of the initial pH and inoculation ratio. Five temperatures were chosen between the optimal enzyme production temperatures (23 °C and 37 °C) of the two bacterial strains, and 30 °C was found to be the best temperature for feather degradation (Additional file 1: Fig. S1c). The keratinase activity peaked at 25 °C. Nevertheless, in line with the results obtained when feathers were degraded by *S. maltophilia* BBE11-1 alone, higher keratinase activity did not correspond to increased degradation, because low temperature decreases the enzymatic activity of *Bacillus licheniformis* BBE11-1.

### Characterization of three degradation systems

**Fig. 2:**
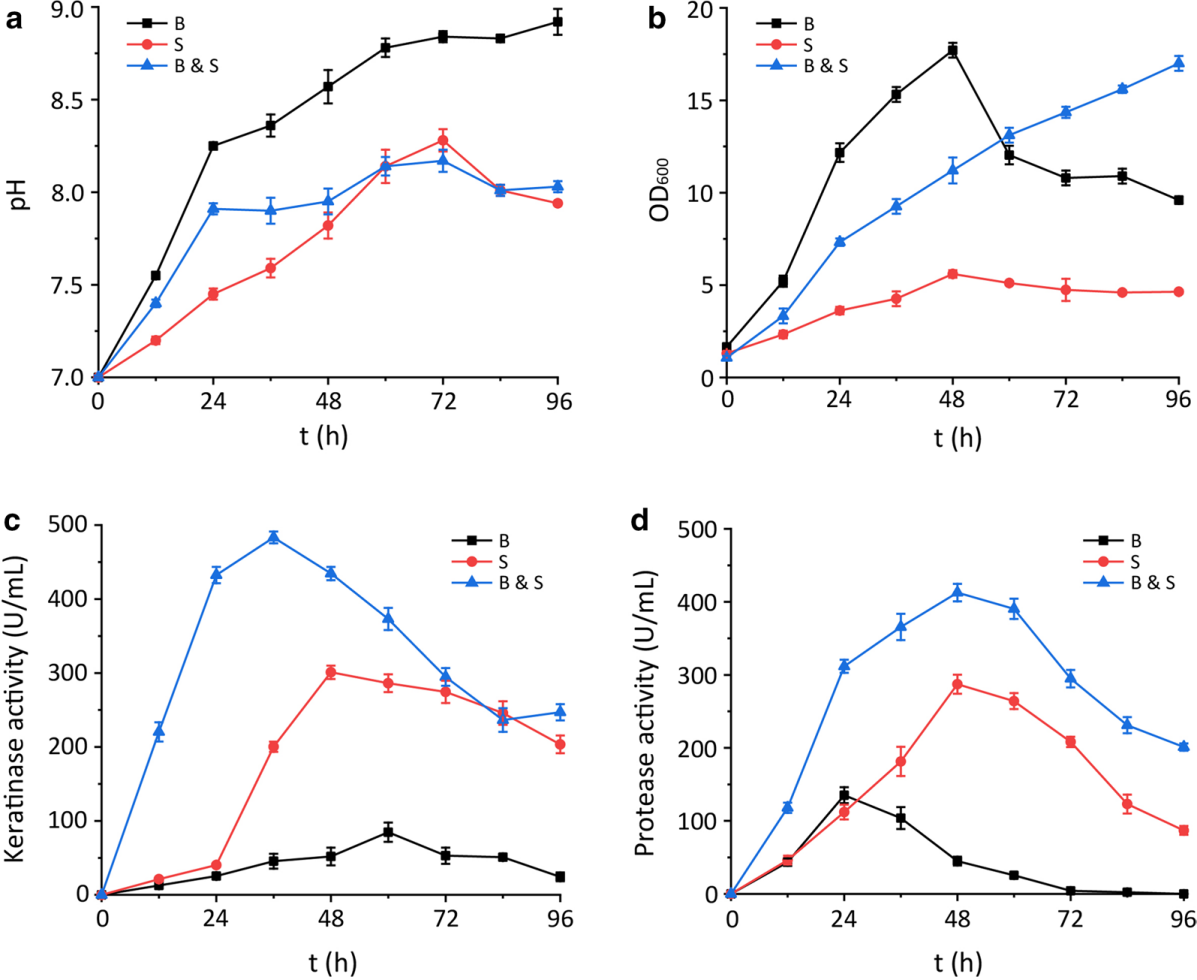
Characterization of three degradation systems. **a** Changes in pH of the degradation systems; **b** changes in cell density of the degradation systems; **c** changes in keratinase activity of the degradation systems; **d** changes in protease activity of the degradation systems. (B: *B. licheniformis* BBE11-1, S: *S. maltophilia* BBE11-1)

Cell density, pH, and keratinase and protease activities were monitored to determine the relation and difference between single-culture degradation and coculture-based degradation. Cocultivation was carried out in the optimal condition (initial pH 7.0, inoculum ratio 1:1, conversion temperature 30 °C). The pH of the degradation system of *B. licheniformis* BBE11-1 was higher than that of the *S. maltophilia* BBE11-1 and coculture system (Fig. 2a). This parameter may have affected the degradation process. The cell density in the *B. licheniformis* BBE11-1 degradation system reached a maximum of 17.71 (OD_600_) at 48 h and then decreased sharply (Fig. 2b). This sharp decrease in cell density was not observed in the degradation system of *S. maltophilia* BBE11-1 and in the coculture degradation system. By contrast, the cell density in the *S. maltophilia* BBE11-1 system was obviously lower than that in the other two degradation systems, indicating that the low growth rate of *S. maltophilia* BBE11-1 limited the extraction efficiency of keratinase and protease. Therefore, coculture of the two bacteria resulted in stable cell growth at the optimum pH.

The keratinase and protease activities in the coculture system were higher than those in the single-culture system, with maximum activities of 483.4 and 412.7 U/mL, respectively (Fig. 2c, d). The trend in keratinase and protease activities in the coculture system was similar to that in the *S. maltophilia* BBE11-1 degradation system, indicating that *S. maltophilia* BBE11-1 played a dominant role in the extraction of keratinase and protease. Nevertheless, keratinase activity in the coculture system substantially increased from 0 to 24 h (Fig. 2c), in contrast to the keratinase activity during the *S. maltophilia* BBE11-1–driven degradation. Keratinase activity was improved because *B. licheniformis* BBE11-1 preferentially degrades feathers and produces nutrients such as amino acids and soluble peptides, which are then utilized by *S. maltophilia* BBE11-1 to accelerate their growth and increase the secretion of keratinase.

Additionally, the keratinase and protease activities in the *S. maltophilia* BBE11-1 degradation system were higher than those in the *B. licheniformis* BBE11-1 system, but the degradation ability was lower. This result indicates that keratin degradation is mediated by the synergistic action of keratinase and a variety of other proteases [34, 35]. Therefore, in the coculture system, *B. licheniformis* BBE11-1 supplied the most complex proteases, while *S. maltophilia* BBE11-1 provided higher keratinase and protease activities (Fig. 2c, d). These factors functioned together to achieve higher degradation efficiency and improved degradative effects on chicken feather waste.

### Co-culture of *B. licheniformis* BBE11-1 and *S. maltophilia* BBE11-1 to degrade chicken feathers in a 3 L fermenter

**Fig. 3:**
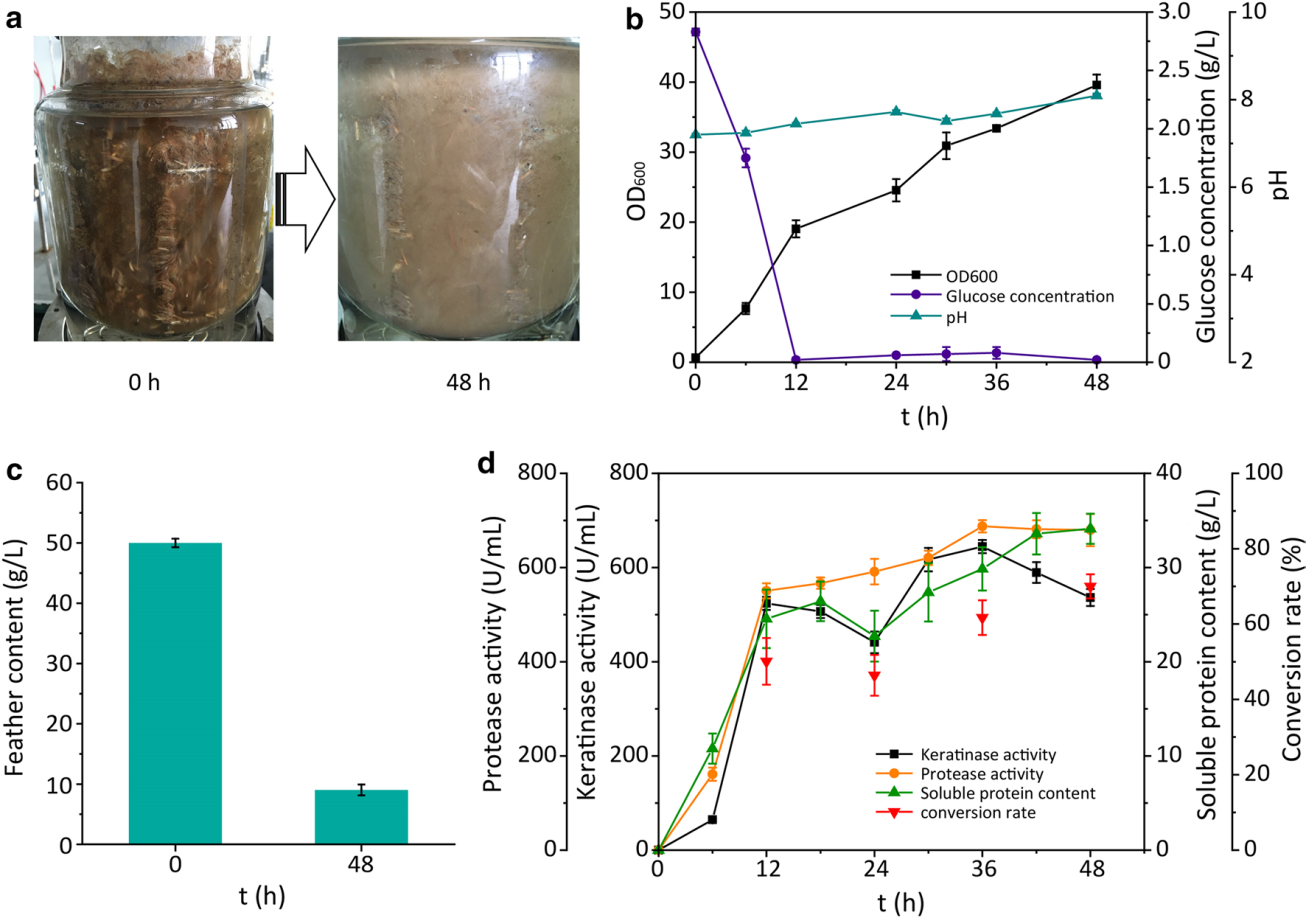
Biodegradation of chicken feather waste in a scale-up 3 L fermenter by cocultivation. **a**, **c** Feather degradation results of cocultivation. **b** Changes in cell density, glucose concentration, and pH in the reaction system. **d** Keratinase activity and protease activity in the process of hydrolysis

**Fig. 4:**
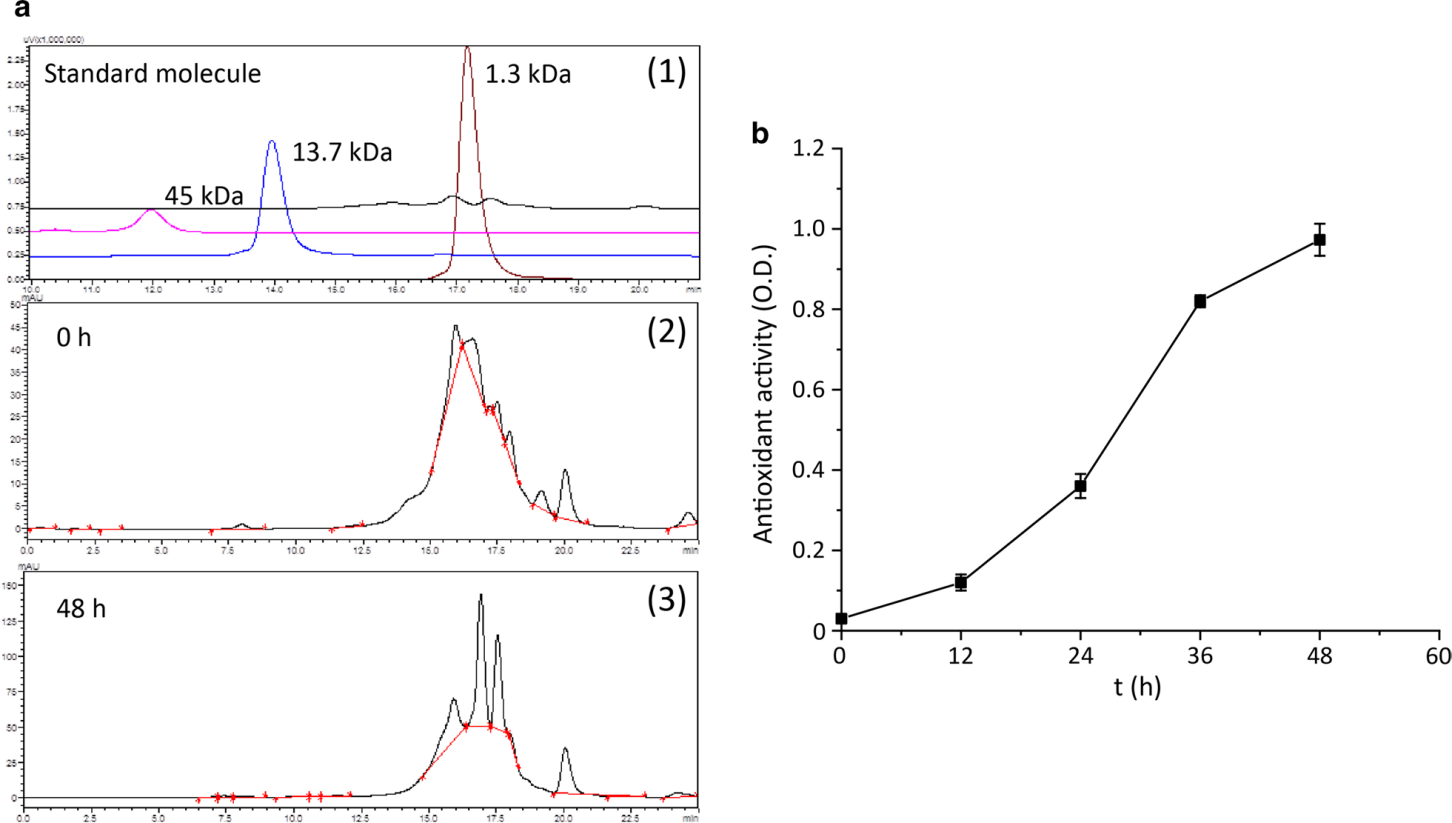
Molecular weight distribution of polypeptides in and antioxidant activity of the hydrolysate. **a** Molecular weight distribution of polypeptides in the hydrolysate. **b** Antioxidant activity of the hydrolysate

Coculture of *B. licheniformis* BBE11-1 and *S. maltophilia* BBE11-1 in shaking flasks significantly improved the efficiency of chicken feather degradation. To further improve the efficiency of chicken feather degradation under coculture conditions, the reaction system was scaled up to a 3 L fermenter with dissolved oxygen control and two-stage temperature control. Unexpectedly, after 48 h of cultivation, nearly all chicken feathers were degraded, with the degradation rate of 81.8%, leaving only the scapus (9.1 g/L) in the culture, which is very difficult to decompose (Fig. 3a, b). The trend in bacterial density was similar to that in the shaking-flask experiment, but the absolute value was doubled, and pH remained stable and gradually approached 8.0 (Fig. 3c). Figure 4d indicates that the keratinase and protease activities increased rapidly in the first 12 h and remained high (approximately 600 U/mL) from 12 to 48 h.

Additionally, cocultivation of *B. licheniformis* BBE11-1 and *S. maltophilia* BBE11-1 in the 3 L fermenter increased the efficiency of degradation of chicken feather waste by reducing the degradation time to 48 h (half of the degradation time in shaking flasks). Therefore, cocultivation of *B. licheniformis* BBE11-1 and *S. maltophilia* BBE11-1 shows an industrial potential for chicken feather waste degradation.

### Characterization of feather hydrolysate

Table 1 shows the changes in the amino acid composition and concentration of hydrolysate samples at different time points of the degradation process in the cocultured batch. The total amino acid content of the hydrolysate reached 895.89 mg/L after 48 h of hydrolysis; the concentrations of tyrosine (Tyr), valine (Val), phenylalanine (Phe), and leucine (Leu) increased 6.6-, 5.5-, 5.4-, and 2.1-fold, respectively, over the original values. These concentrations were much higher than those reported previously (Table 2) [36, 37]. Val, Phe, and Leu are essential amino acids, and Tyr is a conditionally essential amino acid; these amino acids cannot be synthesized in the body [38]. Therefore, hydrolyzed chicken feathers have a great potential for use as feed additives and amino acid production.

**Table 1:** Types and concentrations of free amino acids in feather hydrolysate

Amino acid	Concentration (mg/L)				
	0 h	12 h	24 h	36 h	48 h
Aspartic acid	11.47 ± 0.22	12.20 ± 0.16	20.80 ± 0.92	19.43 ± 0.88	15.55 ± 1.09
Glutamate	46.23 ± 0.18	31.05 ± 0.44	22.33 ± 0.58	22.07 ± 0.13	20.19 ± 0.13
Serine	12.54 ± 1.09	16.97 ± 0.43	14.44 ± 0.18	14.69 ± 0.24	14.61 ± 0.18
Glycine	39.48 ± 0.81	17.99 ± 0.39	12.39 ± 0.11	12.10 ± 0.34	14.34 ± 0.24
Threonine	15.70 ± 0.28	15.43 ± 0.18	14.01 ± 0.83	14.46 ± 0.56	15.74 ± 0.22
Arginine	16.26 ± 0.36	19.02 ± 0.23	17.33 ± 0.16	18.55 ± 0.89	22.13 ± 0.49
Alanine	24.81 ± 0.44	13.71 ± 0.28	10.65 ± 0.58	9.09 ± 0.27	10.05 ± 0.28
Tyrosine	25.64 ± 1.08	54.16 ± 0.66	65.54 ± 0.82	95.14 ± 0.19	171.53 ± 0.42
Valine	37.86 ± 0.21	69.52 ± 0.72	99.29 ± 1.05	123.77 ± 0.25	207.51 ± 0.29
Methionine	23.03 ± 0.14	19.75 ± 0.63	18.94 ± 0.42	15.96 ± 0.54	0.85 ± 0.41
Phenylalanine	33.73 ± 1.04	54.27 ± 1.06	75.82 ± 0.15	98.37 ± 0.91	183.73 ± 0.21
Isoleucine	29.67 ± 0.11	39.80 ± 0.27	48.37 ± 0.19	53.76 ± 0.03	72.96 ± 0.49
Leucine	58.99 ± 0.21	67.84 ± 0.77	73.61 ± 0.31	80.71 ± 1.05	126.61 ± 0.73
Lysine	51.29 ± 0.27	18.36 ± 0.82	15.46 ± 0.25	17.54 ± 0.62	20.94 ± 0.79
Proline	ND	ND	ND	ND	ND
Tryptophan	ND	ND	ND	ND	ND
Cysteine	ND	ND	ND	ND	ND
Histidine	ND	ND	ND	ND	ND
Glutamine	ND	ND	ND	ND	ND
Asparagine	ND	ND	ND	ND	ND

*ND* not detected

**Table 2:** Comparison of amino acid concentrations in feather hydrolysate

Amino acid	Concentration (mg/L)			
	This study (48 h)	*S. maltophilia* BBE11-1 (48 h) [39]	*Xanthomonas sp.* P5 (96 h) [37]	*B. pumilus* GRK (48 h) [36]
Aspartic acid	15.55 ± 1.09	2.05	7.7	1.46
Glutamate	20.19 ± 0.13	5.57	6.7	2.23
Serine	14.61 ± 0.18	0.04	11.9	9.84
Glycine	14.34 ± 0.24	4.05	8.4	7.61
Threonine	15.74 ± 0.22	0.12	24.4	0
Arginine	22.13 ± 0.49	0.03	0	3.55
Alanine	10.05 ± 0.28	0.51	5	0.69
Tyrosine	171.53 ± 0.42	5.33	0	0
Valine	207.51 ± 0.29	2.24	4.7	2.31
Methionine	0 ± 0.41	0.92	31.5	7.24
Phenylalanine	183.73 ± 0.21	14.63	0	0
Isoleucine	72.96 ± 0.49	0.05	0	15
Leucine	126.61 ± 0.73	0.02	4	0
Lysine	20.94 ± 0.79	0.28	0	10.81

Figure 3d indicates that the concentration of soluble peptides in the hydrolysate increased with the degree of hydrolysis of the feathers, reaching 34.1 g/L after 48 h of hydrolysis. The conversion of feathers to soluble peptides and amino acids also reached a maximum of 70.0% at 48 h. The soluble hydrolysate in the cocultured batch was analyzed, and the molecular weight of the polypeptides in the hydrolysate were found to be approximately 1.3 kDa (Fig. 4a). This result indicated that the hydrolysate was mainly composed of short peptides and oligopeptides. These peptides are easily absorbed by humans and animals and have potential applications in food additives, biomedical and cosmetic industry [40]. Additionally, the FRAP assay to revealed that the antioxidant activity of the hydrolysate increased with increased degree of hydrolysis (Fig. 4b). Future work will focus on the identification and separation of the antioxidant components (such as peptides) in the hydrolysate.

## Conclusions

In this study, we developed a method for improving the efficiency of degradation of chicken feather waste using a coculture of *B. licheniformis* BBE11-1 and *S. maltophilia* BBE11-1. This approach solved the limitation of the feather-degrading ability of wild-type strains. Keratinase and protease activities and feather degradation rates of the coculture system was greatly improved compared with those of the single-culture systems. Cocultivation in a 3 L fermenter for 48 h achieved a degradation rate of 81.8% for 50 g/L chicken feather waste. In addition, the microbial chicken feather degradation process is environmentally friendly, and the resulting hydrolysate is enriched with bioactive amino acids and peptides at a conversion rate of 70.0%, which is economical and sustainable for animal feed. Nevertheless, the degradation process is accompanied by bacterial metabolism, which prevents the amino acid content in the feather hydrolysate from reaching the desired high value. Therefore, further research is needed to optimize the conversion rate. Moreover, large number of active polypeptides is produced during the hydrolysis, which are valuable and worthy of careful investigation.

## Methods

### Keratinolytic strains and culture medium

The two chicken feather-degrading strains *B. licheniformis BBE11*-*1* (CCTCC NO. M2011319) and *S. maltophilia* BBE11-1 (CCTCC NO. M2011193) were identified by screening in our previous studies. In this study, *B. licheniformis* BBE11-1 and *S. maltophilia* BBE11-1 were cultured in a chicken feather medium (initial pH 8.0) comprising (g/L): chicken feathers 50, yeast extract 1.5, glucose 3.0, KH_2_PO_4_ 0.7, K_2_HPO_4_ 1.4, NaCl 0.5, and MgSO_4_ 0.1. Individual cultures of *B. licheniformis* BBE11-1 and *S. maltophilia* BBE11-1 were carried out as described previously [39, 41].

### Preparation of chicken feathers and degradation rate calculation

Chicken feather waste was collected from a local poultry market (Wuxi, China), washed with tap water, and dried in an oven at 65 °C for 24 h, and the dried feathers were placed in a Ziploc bag for subsequent analysis. The biodegradation of feathers was carried out in a sterile environment. The feathers were pretreated at 121 °C for 15 min and the subsequent operations were all sterile. The degradation rate of feathers was measured as the change in dry weight before and after degradation. The hydrolysate was passed through a filter paper to remove unhydrolyzed feathers, and the removed feathers were washed several times with deionized water to completely remove the soluble materials and bacteria, followed by drying in an oven at 65 °C for 24 h. The feather degradation rate was calculated using the following formula:

$${\text{Feather degradation rate }}\left( \% \right) \, = \, 100 \, \times {{\left( {{\text{B}} - {\text{A}}} \right)} \mathord{\left/ {\vphantom {{\left( {{\text{B}} - {\text{A}}} \right)} {\text{B}}}} \right. \kern-0pt} {\text{B}}}$$

where B is the dry weight of the feathers before decomposition, and A is the dry weight of the feathers after decomposition.

### Shaking flask experiments

All laboratory-scale degradation experiments were carried out in a 500 mL Erlenmeyer flask. Each flask contained 50 mL of the culture medium supplemented with 50 g/L chicken feather waste. Colonies activated by scribing were inoculated into a 200 mL Erlenmeyer flask containing 50 mL of the Luria–Bertani (LB) medium and were incubated at 37 °C with agitation at 220 rpm for 16 h. Next, 10 mL of the inoculum was transferred to the degradation system. Degradation experiments were initially conducted at 37 °C or 23 °C with agitation at 220 rpm for 96 h; each experiment was repeated three times.

### Optimization of co-culture conditions

Because the two strains show large differences in their initial pH and culture temperature, we optimized the initial pH, culture temperature, and the inoculation ratio for the coculture system. The coculture conditions were optimized by changing the initial pH (7.0, 7.5, 8.0, 8.5, and 9.0), inoculation ratio of *B. licheniformis* BBE11-1 and *S. maltophilia* BBE11-1 (3:1, 2:1, 1:1, 1:2, 1:3), and culture temperature (23 °C, 25 °C, 30 °C, 33 °C, and 37 °C) of the degradation system. Optimization of degradation conditions was evaluated by the degradation rate and keratinase activity. The coculture conditions were optimized using a single factor test, and the first-stage incubation of all experiments was conducted 37 °C for 12 h in order to shorten the growth time of the cells and then switched to the set temperature. The total inoculum was 20% in all experiments.

### Laboratory fermenter batch experiments

The results of laboratory-scale degradation experiments were verified in a 3 L fermenter (BioFlo110, New Brunswick Scientific Co., Edison, NJ, USA) containing 1.5 L of the culture medium with an inoculum volume of 20% of the total volume and 50 g/L chicken feather waste. The degradation processes were started at 500 rpm agitation and a 2.0 vvm air flow rate. Each of the two strains was inoculated at a volume of 10% of the total volume. The initial temperature was 37 °C and was changed to 30 °C until 12 h after fermentation, and the dissolved oxygen level was maintained at 30% by controlling the mixing speed and air volume.

### Keratinolytic and proteolytic activity assay

Throughout the chicken feather degradation experiment, changes in keratinolytic activity and proteolytic activity were monitored for process optimization. The keratinolytic activity assay was conducted as described previously [42] with a minor modification. The reaction system containing 150 μL of 50 mM Gly/NaOH buffer (pH 9.0), 100 μL of 2.5% soluble keratin, and 50 μL of a suitably diluted enzyme solution was incubated at 50 °C for 20 min. The reaction was terminated by adding 200 μL of 4% trichloroacetic acid (TCA) and centrifugation at 8000 rpm at room temperature for 3 min. For the Folin–Ciocalteu method, 200 μL of the supernatant was mixed with 1 mL of 4% Na_2_CO_3_ and 200 μL of the Folin–Ciocalteu reagent at 50 °C for 10 min. The absorbance at 660 nm was measured, and the corresponding enzymatic activity was determined by tyrosine standard curve conversion. All the experiments were repeated three times, and TCA was added to the control group before addition of the enzyme solution. The remaining operations were the same as those in the experimental group. In this study, one unit of keratinolytic activity was defined as 1 μmol tyrosine liberated per minute of substrate conversion.

Proteolytic activity was also determined by the Folin–Ciocalteu method. First, 200 μL of an enzyme solution was mixed with 200 μL of casein dissolved in phosphate buffer and incubated at 40 °C for 30 min, and then 400 μL of 0.4 M TCA was added to terminate the enzymatic reaction. The samples were centrifuged at 8000 rpm at room temperature for 3 min; 150 μL of the supernatant was mixed with 750 μL of 0.4 M Na_2_CO_3_ and 200 μL of the Folin–Ciocalteu reagent at 40 °C for 20 min. The absorbance at 680 nm was measured, and the other parameters were determined as described earlier. One unit of proteolytic activity was defined as 1 μg of tyrosine liberated per minute of casein conversion at 40 °C.

### Antioxidant analysis of chicken feather hydrolysate

The clarified feather hydrolysate was obtained by filtration through eight layers of gauze and centrifugation at 12,000×*g* for 20 min. The antioxidant properties of the chicken feather hydrolysates sampled at different time points were analyzed using the Total Antioxidant Capacity Assay Kit (Beyotime Institution of Biotechnology, Shanghai, China). Specific operational details of the FRAP method were as follows. First, 180 μL of a FRAP working solution was added into each well of a 96-well plate, and then 5 μL of various samples were added to the sample wells, while 5 μL of distilled water was added to the blank wells. Absorbance at 593 nm (A_593_) was measured after incubation at 37 °C for 3–5 min. For the FRAP method, the total antioxidant capacity was expressed as the concentration of a FeSO_4_ standard solution.

### Analysis of amino acids and soluble peptides

Samples were centrifuged at 8000 rpm for 5 min, and then the supernatant was removed, mixed with the same volume of TCA, and incubated at 4 °C for at least 30 min. The mixture was centrifuged, and the supernatant was passed through a 0.2 μm membrane filter. The free-amino-acid composition was determined by high-performance liquid chromatography (Agilent 1260, Santa Clara, CA, USA) with o-phthalaldehyde-9-fluorenylmethyl chloroformate (OPA-FMOC) precolumn derivatization [43]. The concentrations were calculated from the resulting peak areas using an Agilent spectrometry system. The mobile phase used as acetonitrile–methanol. The detector, wavelength, and flow rate were VWD, 338 nm, and 1 mL/min, respectively. The column, temperature, and injection volume were Hypersil ODS-2 (250 × 4.6 mm, 5 μm), 40 °C, and 10 μL, respectively.

The soluble peptides were also determined by high-performance liquid chromatography (Agilent 1260) by comparing the peak time and peak area. The sample was processed in the same manner as in amino acid detection method, except that the same volume of TCA was not required to remove the protein. The obtained soluble peptides were separated on TSK gel G2000SWXL (7.8 × 300 mm) by gradient elution with phosphate buffer as the mobile phase [44]. The detector, wavelength, and flow rate were VWD, 214 nm, and 0.8 mL/min, respectively.

The content of soluble peptides in the hydrolysate was determined using the Bradford method, and the sample was treated in the same manner as in the peptide determination method.

## Additional file

**Additional file 1: Fig. S1.** Optimization of co-culture conditions. (a) Optimization of initial pH; (b) Optimization of the inoculation ratio of *B. licheniformis* BBE11-1 and *S. maltophilia* BBE11-1; (c) Optimization of conversion temperature.

## Data Availability

All data generated or analyzed during this study are included in this published article and its additional files.

## Abbreviations

TCA: trichloroacetic acid
OPA-FMOC: *o*-phthalaldehyde-9-fluorenylmethyl chloroformate
