# Supplementary material for: Effective biodegradation of chicken feather waste by co-cultivation of keratinase producing strains
Source: Microb Cell Fact. 2019 May 18;18:84. doi: 10.1186/s12934-019-1134-9 (PMC6525419; doi:10.1186/s12934-019-1134-9)
Supplement: Supplementary file 1 — Additional file 1: Fig. S1. Optimization of co-culture conditions. (a) Optimization of initial pH; (b) Optimization of the inoculation ratio of B. licheniformis BBE11-1 and S. maltophilia BBE11-1; (c) Optimization of conversion temperature. [file 12934_2019_1134_MOESM1_ESM.docx]

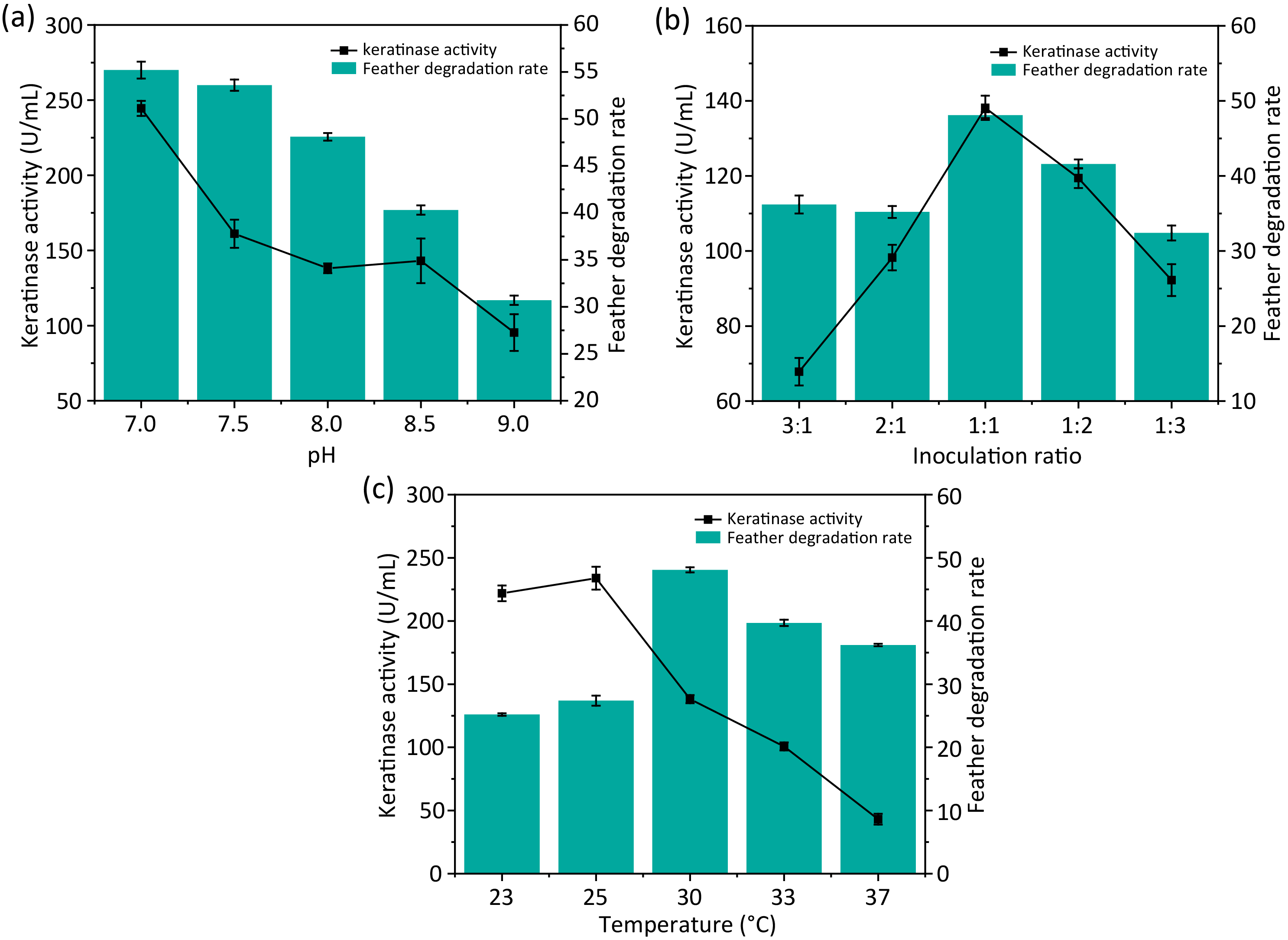


**Fig. S1.** Optimization of co-culture conditions. (a) Optimization of initial pH; (b) Optimization of the inoculation ratio of *B. licheniformis* BBE11-1 and *S. maltophilia* BBE11-1; (c) Optimization of conversion temperature.
